# metaSNV v2: detection of SNVs and subspecies in prokaryotic metagenomes

**DOI:** 10.1093/bioinformatics/btab789

**Published:** 2021-11-17

**Authors:** Thea Van Rossum, Paul I Costea, Lucas Paoli, Renato Alves, Roman Thielemann, Shinichi Sunagawa, Peer Bork

**Affiliations:** Structural and Computational Biology Unit, European Molecular Biology Laboratory (EMBL), 69117 Heidelberg, Germany; Structural and Computational Biology Unit, European Molecular Biology Laboratory (EMBL), 69117 Heidelberg, Germany; Department of Biology, Institute of Microbiology and Swiss Institute of Bioinformatics, ETH Zürich, 8092 Zürich, Switzerland; Structural and Computational Biology Unit, European Molecular Biology Laboratory (EMBL), 69117 Heidelberg, Germany; Structural and Computational Biology Unit, European Molecular Biology Laboratory (EMBL), 69117 Heidelberg, Germany; Department of Biology, Institute of Microbiology and Swiss Institute of Bioinformatics, ETH Zürich, 8092 Zürich, Switzerland; Structural and Computational Biology Unit, European Molecular Biology Laboratory (EMBL), 69117 Heidelberg, Germany; Department of Bioinformatics, Biocenter, University of Würzburg, 97070 Würzburg, Germany; Yonsei Frontier Lab (YFL), Yonsei University, Seoul 03722, South Korea

## Abstract

**Summary:**

Taxonomic analysis of microbial communities is well supported at the level of species and strains. However, species can contain significant phenotypic diversity and strains are rarely widely shared across global populations. Stratifying the diversity between species and strains can identify ‘subspecies’, which are a useful intermediary. High-throughput identification and profiling of subspecies is not yet supported in the microbiome field. Here, we use an operational definition of subspecies based on single nucleotide variant (SNV) patterns within species to identify and profile subspecies in metagenomes, along with their distinctive SNVs and genes. We incorporate this method into metaSNV v2, which extends existing SNV-calling software to support further SNV interpretation for population genetics. These new features support microbiome analyses to link SNV profiles with host phenotype or environment and niche-specificity. We demonstrate subspecies identification in marine and fecal metagenomes. In the latter, we analyze 70 species in 7524 adult and infant subjects, supporting a common subspecies population structure in the human gut microbiome and illustrating some limits in subspecies calling.

**Availability and implementation:**

Source code, documentation, tutorials and test data are available at https://github.com/metasnv-tool/metaSNV and https://metasnv.embl.de.

**Supplementary information:**

Supplementary data are available at *Bioinformatics* online.

## 1 Introduction

Metagenomic single nucleotide variant (SNV) calling has proven useful in many contexts, such as tracking strains between habitats (Schmidt *et al.*, 2019) and identifying subspecies in the human microbiome (Costea *et al.*, 2017b). Subspecies are a useful taxonomic resolution because they often have distinct habitats and/or functional traits (Monroe, 1982; Patten, 2015; Van Rossum *et al.*, 2020). For example, *Bifidobacterium longum* has subspecies associated with infants (subsp. *infantis*) and nonhuman animal hosts (subsp. *suis*) (O’Callaghan *et al.*, 2015) and subspecies (‘phylotypes’) in *Escherichia coli* are associated with differences in habitat, antibiotic resistance and pathogenicity (Bailey *et al.*, 2010). While species-specific microbiome approaches exist (Karcher *et al.*, 2020; Milani *et al.*, 2014), no software yet exists to broadly delineate subspecies from metagenomic data. Many tools characterize population-level diversity [MIDAS (Nayfach *et al.*, 2016), metaSNV v1 (Costea *et al.*, 2017a), POPGENOM (Sjöqvist *et al.*, 2021), inStrain (Olm et al., 2021), StrainPhlAn (Truong *et al.*, 2017)] and/or recover haplotypes or strains from metagenomes [DESMAN (Quince *et al.*, 2017), ConStrains (Luo *et al.*, 2015), InStrain (Olm *et al.*, 2021), strainGEMS (Tan *et al.*, 2019)], with varying definitions of strains (Van Rossum *et al.*, 2020). However, none of these tools provide robust clustering of population diversity for data-driven identification of subspecies. Here, we present metaSNV v2, which supports detection of SNVs and population genetic analysis, including population subspecies identification and profiling.

## 2 Tool description

metaSNV v2 builds on metaSNV v1 (Costea *et al.*, 2017a), which identifies SNVs using SAMtools mpileup (Li, 2011) and mappings of short read metagenomic data against species-specific genomic references (BAM files). Dissimilarities between metagenomes based on SNV profiles are then calculated for each species. In metaSNV v2, the SNV postprocessing is improved and extended in various ways. For example, a subspecies module has been added (detailed below), SNV filtering has been parallelized and estimates of purifying selection have been added. metaSNV v2 now natively supports a larger database of high quality reference genomes [based on ProGenomes2 (Mende *et al.*, 2020)]. Comprehensive user documentation has been newly developed, including a detailed description of the method (see GitHub repository).

The most significant functionality added to metaSNV v2 is the ‘subpopr module’. In short, this module detects ‘population subspecies’ (Van Rossum *et al.*, 2020) by calculating SNV-based dissimilarities between a species’ populations across metagenomic samples and assessing whether these populations form distinct clusters. Subspecies detection is performed for each species in the reference database, can be run in parallel, and follows the steps described below using the output of metaSNV v2’s SNV calling and filtering.

For each species, a ‘discovery subset’ of metagenomes is selected wherein the species is abundant and its population likely contains a single subspecies. The latter criterium is satisfied if a metagenome contains minimal internal allele variation relative to the SNV variation across all sampled metagenomes (e.g. at least 80% of the dataset-wide species SNVs have the same allele in over 90% of reads in a metagenome). This criterium has been previously used (Costea *et al.*, 2017b), and since conceptualized as ‘quasi-phaseability’ (Garud *et al.*, 2019). The default parameters target subspecies but can be altered to detect subpopulations defined in a more stringent or lenient way (i.e. with varying levels of diversity between and within them) (discussed in Supplementary Information S1). If no metagenomes meet the discovery subset criteria for a species, then subspecies cannot be detected for that species.

This discovery subset of metagenomes is then tested for robust clustering into subspecies based on their SNV-profile dissimilarities. Clustering confidence is assessed using repeated subsampling and the Prediction Strength algorithm (Tibshirani and Walther, 2005), which yield confidence scores for both the number of clusters (subspecies) and their compositions. Distinctive genotyping SNV alleles are then identified per subspecies. These alleles can be used to estimate the relative abundance of each subspecies in any metagenome, including from later independent studies. Subspecies-specific genes are detected by testing for correlations in abundance between genes and subspecies across metagenomes. All results are summarized in plain text and html reports, with embedded plots and statistical test results.

## 3 Results

To demonstrate the functionality of metaSNV v2, we analyzed 7523 human fecal metagenomes from adults and infants from 27 countries for 70 prevalent and abundant gut species, of which 42 stratified into multiple subspecies (Supplementary Information S1). To compare this to a previous subspecies estimate (Costea *et al.*, 2017b), a ‘reduced’ analysis of 1663 adult-only, geographically limited metagenomes was run and 81% (44/54) agreement in subspecies presence was observed with the previous study (Supplementary Information S1). In the ‘reduced’ and the full (*N* = 7523) datasets, 83% (44/53) of species had the same number or lack of subspecies, illustrating the dependence on the input set of metagenomes as in many habitats bacterial populations are still insufficiently covered. For example, subspecies were not detected for *B.longum* in the adult-only dataset, but its subspecies associated with infants [ssp. *infantis* (Sela *et al.*, 2008)] was detected in the analysis which included infants. As a further usage demonstration, metaSNV v2 was run on 288 marine metagenomes (Sunagawa *et al.*, 2015). Out of 10 species with sufficient prevalence and abundance, subspecies were found for two species, each with distinctive geographic enrichment (Supplementary Information S2).

The core SNV-calling code has not been altered in metaSNV v2 and the resource usage statistics and software comparison from the previous release still apply (Costea *et al.*, 2017a). Many tools delineate strains and/or build within-species phylogenetic trees from metagenomic data [listed above and reviewed in Van Rossum *et al.* (2020)]. These tools have different concepts of strains, yet all have a fundamentally different resolution than that of subspecies. This difference precludes a direct comparison to these tools for subspecies classifications.

To validate the results from subspecies calling in metaSNV v2, we used simulated and real data from species with known subspecies. A metaSNV v2 analysis of simulated metagenomes (*N* = 543) composed from mixtures of 540 *E.coli* genomes representing 11 phylogroups (Waters *et al.*, 2020; analogous to subspecies) accurately recovered nine subspecies, with the remaining two closely related phylogroups merged into one subspecies (Supplementary Information S3). After pooling these two phylogroups, genomes were accurately classified to their expected phylogroups in 98% of cases, with all misclassifications to a closely related phylogroup. Though subspecies classifications are not produced by other tools, metaSNV v2 and StrainPhlAn both output SNV-based metagenome similarities, which were well correlated for these simulated metagenomes (Spearman *R* > 0.8, *P* < 2.2e−16, Supplementary Information S3).

*In silico E.coli* and *B.longum* metagenomes were used to validate the subspecies identified from the metaSNV v2 analysis of 7523 fecal metagenomes. Results were as expected for 99% of *E.coli* metagenomes (318/321) and all *B.longum* metagenomes (12/12). Further, as expected, the subspecies corresponding to *B.longum* susbp. *infantis* was almost exclusively seen in infants (61/62) and contained a marker gene for the subspecies, sialidase (Blanco *et al.*, 2020; Supplementary Information S3).

## 4 Conclusions

metaSNV v2 features a number of technical improvements over its predecessor in performing within-species SNV calling on metagenomic samples and expanded functionality for SNV-based population genetic analyses, including subspecies and respective differential gene content detection. This supports comparisons of samples at a taxonomic resolution that is derived from the structure of the data itself, enabling hypothesis generation for phenotypic associations and niche adaptation.

## Supplementary Material

btab789_supplementary_data
