# Supplementary material for: metaSNV v2: detection of SNVs and subspecies in prokaryotic metagenomes
Source: Bioinformatics. 2021 Nov 17;38(4):1162–4. doi: 10.1093/bioinformatics/btab789 (PMC8796361; doi:10.1093/bioinformatics/btab789)
Supplement: btab789_supplementary_data [file btab789_supplementary_data.zip › S1_Human.pdf]

SNV profiling of human faecal metagenomes across age and geography finds subspecies are common but infant-specificity is rare

This document describes the results of an analysis run with metaSNV v2.

Supplementary tables and data are available on FigShare:
https://doi.org/10.6084/m9.figshare.15131400

Abstract ... *1*
Introduction ... *1*
Methods ... *2*
Dataset ... **2**
SNV calling ... **2**
Subspecies detection ... **2**
Parameter sensitivity analysis ... 3
Comparison across datasets ... 3
Subspecies gene content detection ... 3
Subspecies associations with host data ... 3
E. coli phylogroup simulation for validation ... **4**
B. longum subspecies simulation for validation ... **4**
Results ... *5*
Species profiles and within-species diversity ... **5**
Impact of parameter choice on subspecies calling ... **7**
Prevalence of polytypic species ... **8**
Validation using E. coli genomes ... **9**
Validation using B. longum genomes ... **10**
Subspecies enrichment across age and geography ... **11**
Subspecies gene content ... **13**
Impact of population sample on subspecies detection ... **14**
Discussion ... *15*
Comparison to subspecies in literature ... **15**
Subspecies in Streptococcus thermophilus ... **15**
Effect of sample population ... **16**
Limitations ... **16**
Conclusions ... *17*
References ... *17*

Abstract

Within-species variation is a major consideration when studying the human gut microbiome. Despite species being the most common taxonomic level being studied in metagenomic data, a lot of functional variability can exist between clades within the same species. Often, these differences are the result of adaptation to different niches. Many species are seen in both infants and adults, despite their host physiology, diet, and overall microbiome composition being quite different. One species is well known to have infant-associated subspecies (*Bifidobacterium longum* subsp. infantis), with appropriate niche-targeting genes (i.e. for breast milk metabolism). How common are infant-associated subspecies? In the analysis described below, we detected subspecies in faecal metagenomes from 7523 individuals, including adults and infants, from 27 countries. We found only two species with infant-associated subspecies: *B. longum*, as expected, and *Streptococcus thermophilus.* We also found an expanded set of subspecies in adults that are geographically associated. Overall, we found that subspecies are common in gut microbial species, but we did not find evidence that distinct infant-adapted subspecies are common.

Introduction

Conspecific subpopulations of cells with limited gene flow (e.g. due to spatial separation) can eventually diverge into distinct population “subspecies” [1–3]. During this divergence, subspecies acquire different phenotypic potentials, which often reflect the selective forces that shaped their

evolution. For example, *Bifidobacterium longum* has subspecies associated with infants (subsp. *infantis*) and non-human animals (subsp. *suis*) hosts[4] and subspecies ("phylotypes") in *Escherichia coli* are associated with differences in habitat, growth rates, antibiotic resistance, and pathogenicity[5].

While subspecies can cooccur, they must also dominate spatial or temporal subdivisions within the species' range[6]. These subdivisions are likely similar niches and can be geographically distributed, such as different areas in the mouth across the global human population. Subspecies can be operationally defined based on shared (within-subspecies) and distinctive (between subspecies) single nucleotide variants (SNVs), as illustrated for a set of human gut and oral bacterial species from metagenomes[7].

While many tools exist to profile strains in metagenomes, albeit with differing operational definitions of strains (reviewed in [3]), detecting subspecies from large metagenomic data in a high-throughput manner, across thousands of samples and species, has not been supported. Here, we demonstrate how metaSNV v2 can describe subspecies from SNVs identified from metagenomic data. The line between subpopulations and subspecies is not well defined in microbiology and metaSNV v2 allows the user to select parameters to target different segments of this spectrum. Here, we aimed to detect infraspecific subpopulations that have limited inter-subpopulation gene flow, which reflects a definition of subspecies [1–3].

Methods

Dataset

Metagenomic samples were downloaded from ENA from 66 public studies. These samples came from 27 countries and the full age spectrum, with most samples coming from infants or adults (**Table 2**, **Figure 1**). A list of all samples, their sources, and their metadata is available in **Suppl. File S1**.

SNV calling

Metagenomic data from human faeces was gathered from public data for 7523 individuals from 66 studies (**Suppl. File S1**). One sample per individual was used. Metagenomic reads were quality filtered; nucleotide calls with a Phred score of under 25 were removed from the 3' end and reads with less than 45 nucleotides remaining were discarded. Species profiles for each sample were compiled using mOTUs2.5[8]. Metagenomic reads were mapped against 71 species reference genomes (**Suppl. File S2**) from Progenomes2[9] using BWA[10] and ngless[11]. If there were multiple representative genomes in a Progenomes2 species cluster, then the longest genome was used. Read mappings were only kept if the read mapped uniquely across all genomes with a minimum match length of 45bp and minimum percent identity of 97%. Resultant BAM files were used to call SNVs using metaSNV v2 which uses samtools[10,12]. Filtering of metaSNV v2 results was performed to keep only those species that were seen in at least 50 samples with at least 40% genome horizontal coverage and a mean depth of coverage of 5 per sample. Default filtering of SNVs was performed, keeping only those SNVs that were supported by at least 5 reads per sample and whose locus was covered in at least 50% of samples per species.

Subspecies detection

Filtered SNVs were used to calculate dissimilarities between samples using Manhattan distance calculation. Clusters among the samples were detected using metaSNV v2's subpopr module. Parameters were used that are appropriate for detecting subpopulations that correspond to subspecies in the human gut metagenome[7], namely, samples were selected for subspecies detection if at least 80% of their SNV loci had a major allele with frequency greater than 90% ("fixReadThreshold" of 0.1, "fixSnvThreshold" of 0.8 and "genotypingThreshold" of 0.8). The default

values for all other parameter were used. metaSNV v2's subpopr module also produced genotyping SNVs for each subspecies and a profile of subspecies abundances.

Parameter sensitivity analysis

To illustrate the impact of parameter selection on the clustering results, cluster detection was run with a range of parameter values, from 0.65 to 0.95 for "fixSnvThreshold" and 0.35 to 0.05 for "fixReadThreshold". The parameter for identification of genotyping SNVs was not altered here as genotyping was not performed for this parameter sensitivity analysis.

Comparison across datasets

A subset of 1663 BAM files were taken to use as a "reduced" dataset (**Suppl. File S1**). In this "reduced" dataset, we used only adult samples from 9 countries (Austria, China, Denmark, France, Germany, Kazakhstan, Spain, Sweden, USA). All analyses were performed as described above. When comparing results from different subspecies analyses, the number of clusters in the results can vary based on minor differences in the Prediction Strength when the value is close to the threshold (e.g. 0.79 would fail and 0.80 would pass). To avoid noise from this cut-off, clustering results were considered to match if they had a matching number of clusters within a window of Prediction Strength values from 0.75 to 0.85.

Subspecies gene content detection

Gene abundance profiles were compiled per sample by mapping each sample against a set of species' pangenomes, selected to reflect the species with subspecies in this study. Metagenomic reads were mapped against species pangenomes[13] using BWA[10] and ngless[11]. Reads were kept if they mapped uniquely to a gene with a minimum match length of 45bp and minimum percent identity of 97%. Relationships between genes and COG categories were produced by eggNOG5[14] and obtained from the pangenomes, with species provenance information maintained. Gene abundances were normalized to sum to one per sample. Gene profiles were compiled for 6494/7523 samples.

Gene abundance profiles were correlated with species and subspecies abundance profiles in metaSNV v2's subpopr module. Correlations were kept if Spearman correlation R was greater than 0.6 and if Pearson correlations using log10 transformed values had R greater than 0.8.

Enrichment in high level COG categories was calculated for species and subspecies-specific data separately. For each species, the number of correlated genes per COG category was normalized by dividing it by the number of correlated genes for that species. The resultant proportion of genes per species for each COG category was then compared between the species and subspecies-specific data using R[15] with the Wilcox test and p values were controlled for false discovery rate using the Benjamini Hochberg method[16]. COG categories

Subspecies associations with host data

Subspecies associations with metadata (age category, country, and BMI category) were tested using a Fisher's Exact Test on two-dimensional contingency tables using R[15] with the fisher.test function and p values were controlled for false discovery rate using the Benjamini Hochberg method[16]. Categories were tested for association if they had at least 5 samples. Samples were classified as being dominated by a subspecies if they had over 90% relative abundance of that subspecies within the species. To avoid findings that were confounded between age and country, country and BMI associations were only tested among adults. Age was only tested for association using samples from countries that had subjects from more than one age category (e.g. Bangladesh only had child samples so these samples were not included in the testing of age associations). Similarly, BMI associations were only tested using samples from countries that had subjects from more than one BMI category.

E. coli phylogroup simulation for validation

Metagenomic reads were simulated from 540 genomes from *E. coli* using ART_Illumina Version 2.5.8 with read length of 150, genome masking 'N' cut-off frequency of 1 in 150, fold coverage of 100x, and mean fragment length of 400 with standard deviation of 10. The 540 genomes and their phylogroup (analogous to subspecies) classifications are listed in **Suppl. File S8**. There are two main approaches to classify E coli genomes into phylogroups: Clermont typing based on marker gene presence, which generally recognises 7 phylogroups, and whole genome based[2], which recognises 17 phylogroups. This difference is both from subdivision and addition. For example, the genome method breaks up type “B2” into “B2-1” and “B2-2”, and type “D” into “D1”, ”D2”, and ”D3”, and it also has extra groups, such as “Shigella1” and “Shigella2”. For our analysis, we used genomes where both methods agreed on the phylogroup classification. Classifications were used from published data[2], where they were made using EzClermont[3] and Mash[4].

For each sample, *E coli* genome abundances were designed to reflect one community type: “1_PureGenome”, reads from one genome, and “2_PureSubspecies”, reads from multiple genomes from the same phylogroup.

To create these samples, we simulated reads with 20x depth of coverage from 540 *E coli* genomes (46 to 50 per phylogroup, based on availability), then sampled a defined number of reads from each genome’s read set according to the genome’s abundance in the simulated community (**Figure 6a**). This created 543 simulated metagenomic samples (columns in **Figure 6a**).

Subspecies abundances were profiled using metaSNV v2 based on the subspecies’ genotyping SNVs. Reads were mapped against the same reference database as used above and metaSNV v2 was used to call SNVs. The abundances of *E. coli*’s genotyping SNVs (identified above) were then measured in the *in silico* samples and used to calculate subspecies classifications.

B. longum subspecies simulation for validation

Metagenomic reads were simulated from 12 genomes from *Bifidobacterium longum* using ART_Illumina Version 2.5.8 with read length of 150, genome masking 'N' cut-off frequency of 1 in 150, fold coverage of 100x, and mean fragment length of 400 with standard deviation of 10. The 12 genomes and their taxonomic classification are listed in **Table 1**. The reads from each genome were treated as samples and subspecies abundances were profiled using metaSNV v2 based on the subspecies’ genotyping SNVs. Reads were mapped against the same reference database as used above and metaSNV was used to call SNVs. The abundances of B longum’s genotyping SNVs (identified above) were then measured in the *in silico* reads and used to calculate subspecies classifications.

Subspecies from O’Callaghan et al. 2015[4]	Subspecies name	RefSeq Genome Assembly ID
A	longum	GCF_000007525
A	longum	GCF_000008945
A	longum	GCF_000196555
B	infantis	GCF_000269965
B	infantis	GCF_001281305
B	infantis	GCF_000730125
C	“C”	GCF_000092325
C	“C”	GCF_000730205
C	“C”	GCF_000410595
D	suis	GCF_000421385
D	suis	GCF_000741625

D	suis	GCF_000771285

Table 1. Bifidobacterium longum genomes used in subspecies validation.

Results

Species profiles and within-species diversity

Samples gathered were from 66 studies, including samples from 27 countries and the full age spectrum, with most samples coming from infants or adults (**Table 2**, **Figure 1**). Some age-country pairs were confounded, for example, samples from Israel and Mongolia were only from adults and samples from Bangladesh were only from children. A list of all samples, their sources, and their metadata is available in **Suppl. File S1**.

Age category	Year range	Number of subjects
Infant	[0 – 1]	1390
Child	]1 – 10]	104
Teen	]10 – 18]	64
Adult	]18 – 70]	4989
Elder	>70	624
Unknown		352

Table 2. Composition of the study dataset. Number of subjects per age group. One sample per subject was used.

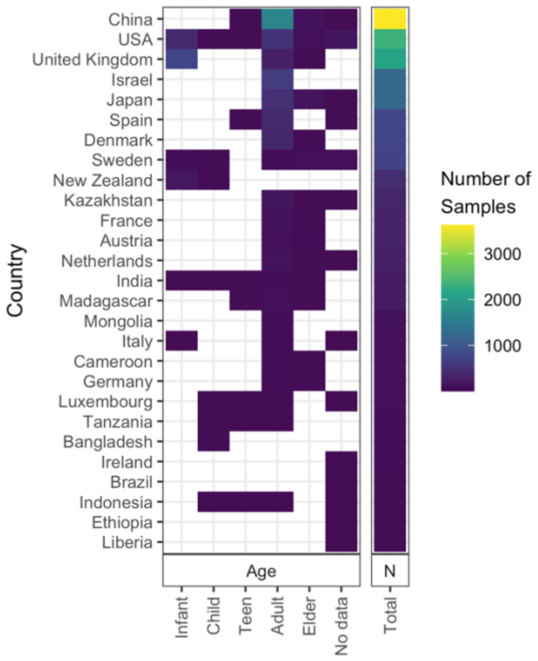

Figure 1. Composition of the study dataset. Number of subjects per country and age group. One sample per subject was used.

Approximately half of the community abundance in these samples is represented by the 71 species used here (mean = 49%, median = 50%, 1st quartile = 34%, 3rd quartile = 65%). Most of these species were seen in adults and infants (**Figure 2**). Of these 71 species, 70 species had sufficient prevalence

and horizontal and vertical coverage in at least 100 samples to have SNVs called. The mean number of samples per species was 1013 (median = 818, 1st quartile = 403, 3rd quartile = 1419, min = 100; max = 4300).

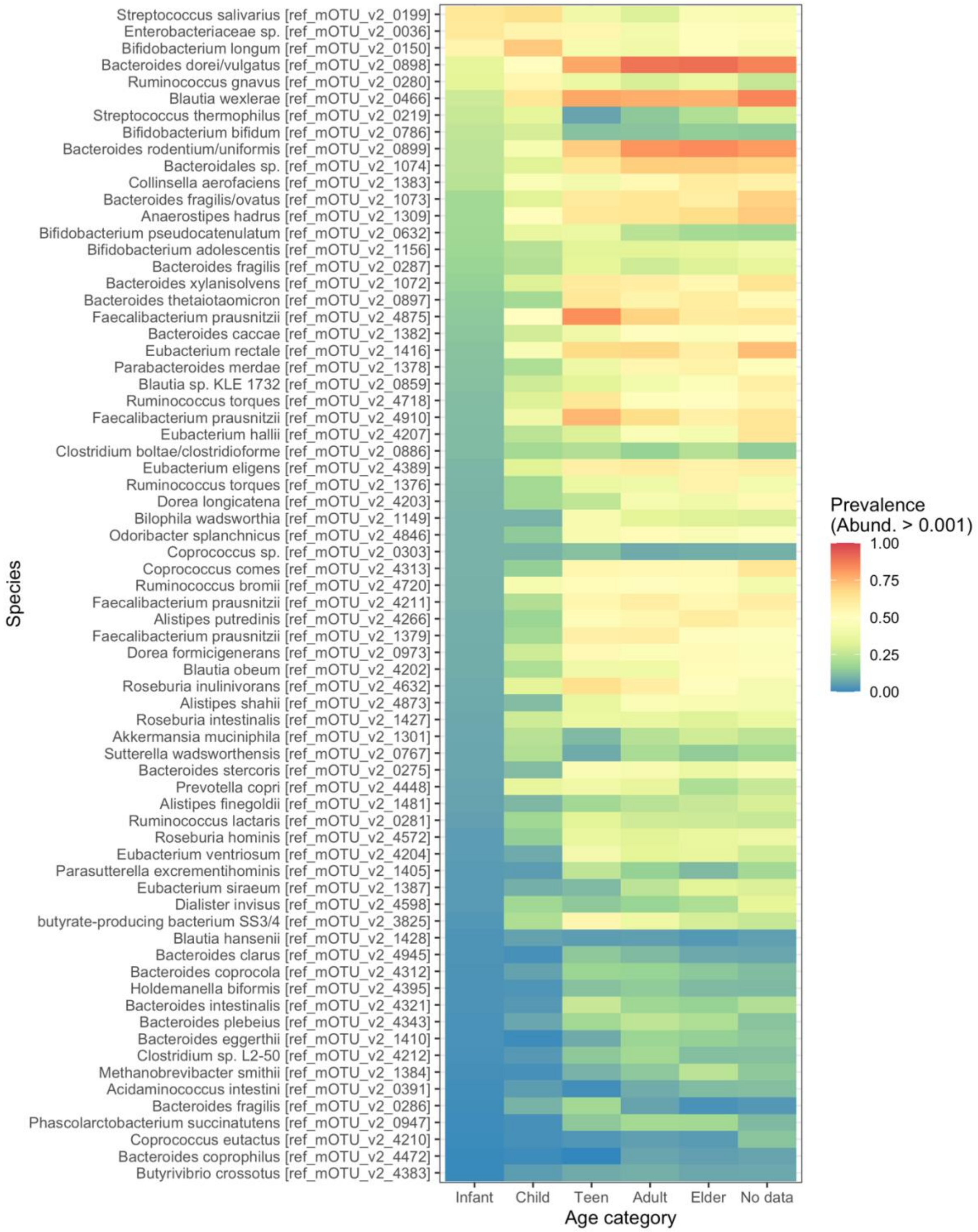

Figure 2. Species prevalence across age categories. A species was considered present if it was in a sample with at least 0.1% abundance. Species are ordered by prevalence in infants.

Across all species and samples, 7,664,864 SNVs were called, with 109,498 mean per species (median = 111,925, 1st quartile = 76016, 3rd quartile = 129151) (**Suppl. File S3**). Two species had less than 10 SNVs called (Bilophila wadsworthia (665950) and [Ruminococcus] torques (1408428)), all others had more than 26,000. The number of SNVs called per species was weakly correlated with the number of samples present (Spearman correlation R=0.3, p value = 0.01) and with the median relative abundance of the species in the samples where it was present (Spearman correlation R=0.24, p value = 0.04).

Impact of parameter choice on subspecies calling

A repeated analysis of this dataset using different values for subpopulation detection parameters was performed to illustrate the impact of parameter choice (**Figure 3**). This analysis focused on the two most biologically relevant parameters: the minimum percentage of SNVs that need to be roughly homologous within a sample (hs) and the maximum proportion of reads that can have a non-major allele for a SNV to be considered roughly homologous (hr). When the confidence in the number of subpopulations (clusters) was rated “high”, parameter choice usually had little effect on the number of clusters called per species, with no difference in 78% of species and a difference of +/-1 in 16% of species, (13/64 and 10/64, respectively). In half of these cases, the difference was seen due to only one parameter pair. More variability was seen when looking at “medium” and “low” confidence clustering calls, with 39% and 80% of species having variability among the number of subpopulations called (23/59 and 24/30 species, respectively). Parameter choice also affected the number of species that had high confidence calls but high confidence clustering calls were always the most common (**Figure 4**). If only considering high confidence calls, then parameter choice is unlikely to heavily impact your results, but it will change the number of species you have results for and, because these parameters represent characteristics of the subpopulations you will identify, the underlying biology that the results describe.

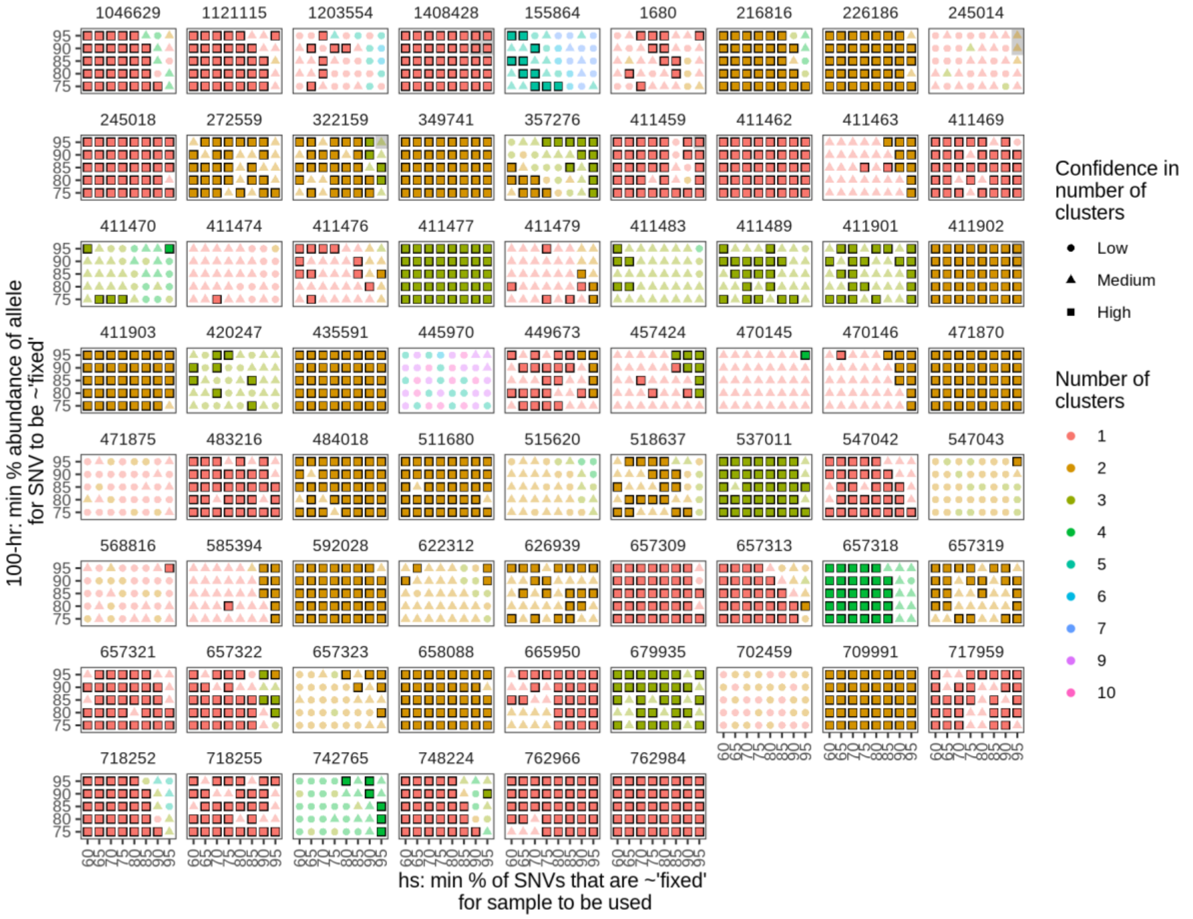

Figure 3. Effect of parameter choice on the number of subpopulations detected. Colours represent the number of subpopulations (clusters). Shapes represent the confidence in each clustering call. High confidence cluster calls are emphasized with black outlined, full opacity squares. Grey backgrounds indicate less than the recommended minimum of 100 samples.

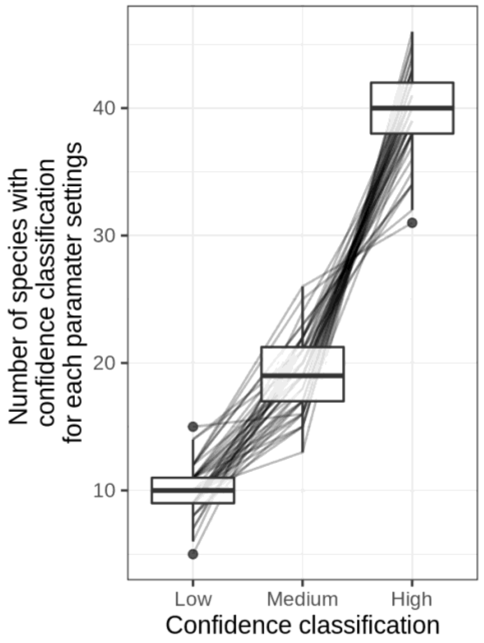

Figure 4. Effect of parameter choice on the number of species with a "high confidence" classification for the number of subpopulations called. Each point is the number of species with this confidence level for one parameter set. Lines connect the same parameter settings. High confidence clustering calls are the most common in this study.

Prevalence of polytypic species

About half of the species tested had detectable subspecies (34/69) (**Suppl. File S4**). If only considering high confidence clustering classifications, then this number stays around 50% (20/41) (**Figure 5A**). Confidence in cluster classification was not associated with sample number in this dataset, suggesting that enough samples were present for robust findings for this population (**Figure 5B**). The number of subspecies found within each species was also not associated with sample number (**Figure 5C**). Genotyping SNVs could be identified for 30/34 polytypic species (**Suppl. File S9**).

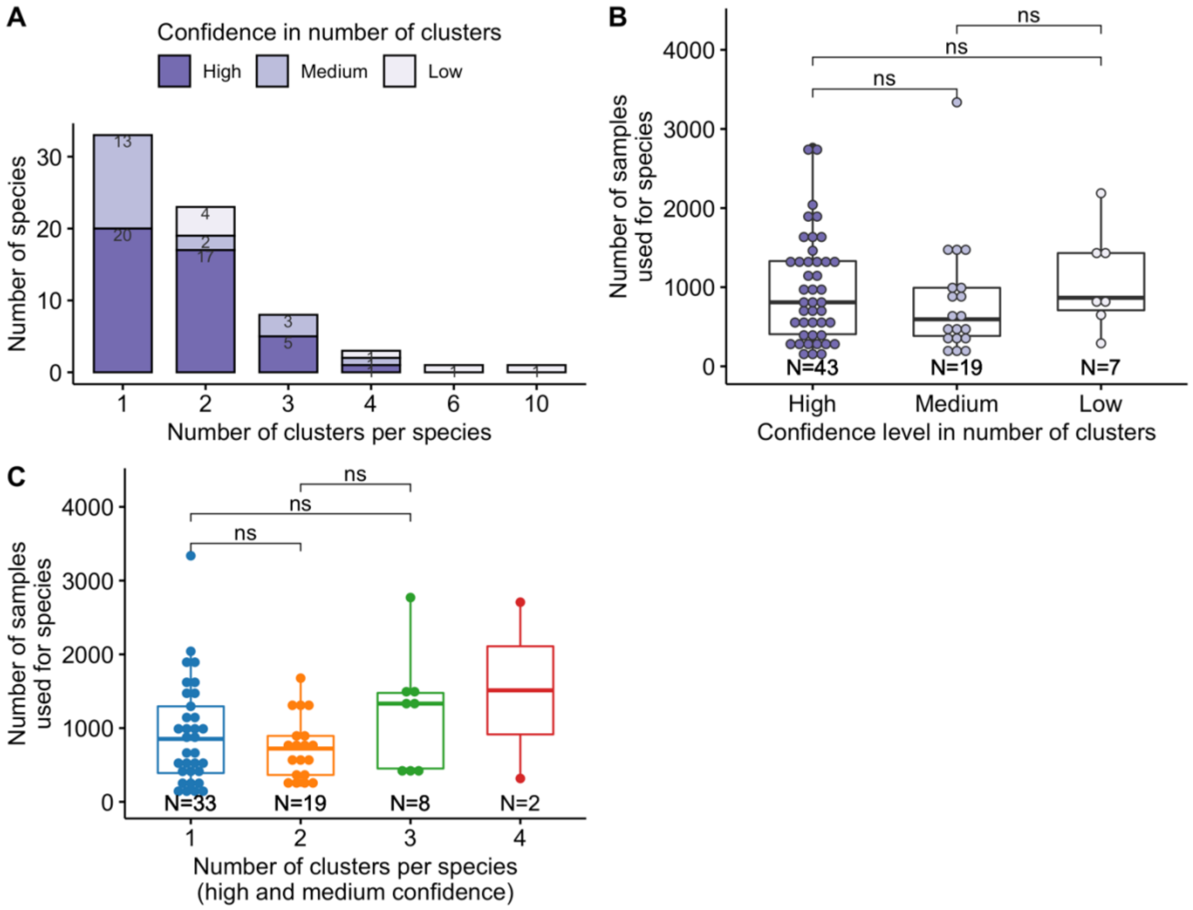

Figure 5. Prevalence of subspecies (clusters) by confidence. (A) Number of subspecies identified per species, coloured by confidence classification. (B) Number of samples per species versus the confidence classification in the number of subspecies (clusters), showing that there is no relationship (Wilcox test). (C) Number of samples per species versus number of subspecies per species, using the high and medium confidence results. More subspecies were not detected in the species that had more samples. N.S. = not significant, p value >0.05.

Validation using E. coli genomes

E. coli genomes can be classified into to phylogroups[17], which are analogous to subspecies. To assess the accuracy of the identified subspecies in this study, *E. coli* genomes with known phylotypes were classified to this study's subspecies using the genotyping SNVs identified by metaSNV v2. Samples of simulated (meta)genomic reads with known phylogroup membership were profiled using the genotyping SNVs produced by metaSNV v2 in this study.

Using 540 genomes from 11 phylogroups, we tested two types of *in silico* generated samples: "1_PureGenome" samples (N=152), where reads are all from one genome, and "2_PureSubspecies" samples (N=169), where reads are from multiple genomes from the same phylogroup (**Figure 6a**). Each sample is expected to have one distinct subspecies classification.

There are two main approaches to classify E coli genomes into phylogroups: Clermont typing based on marker gene presence, which generally recognises 7 phylogroups, and whole genome based[18], which recognises 17 phylogroups. This difference is both from subdivision and addition. For example, the genome method breaks up type "B2" into "B2-1" and "B2-2", and type "D" into "D1", "D2", and "D3", and it also has extra groups, such as "Shigella1" and "Shigella2". For our analysis, we used genomes where both methods agreed on the phylogroup classification. Classifications were used from published data[18], where they were made using EzClermont[19] and Mash[20].

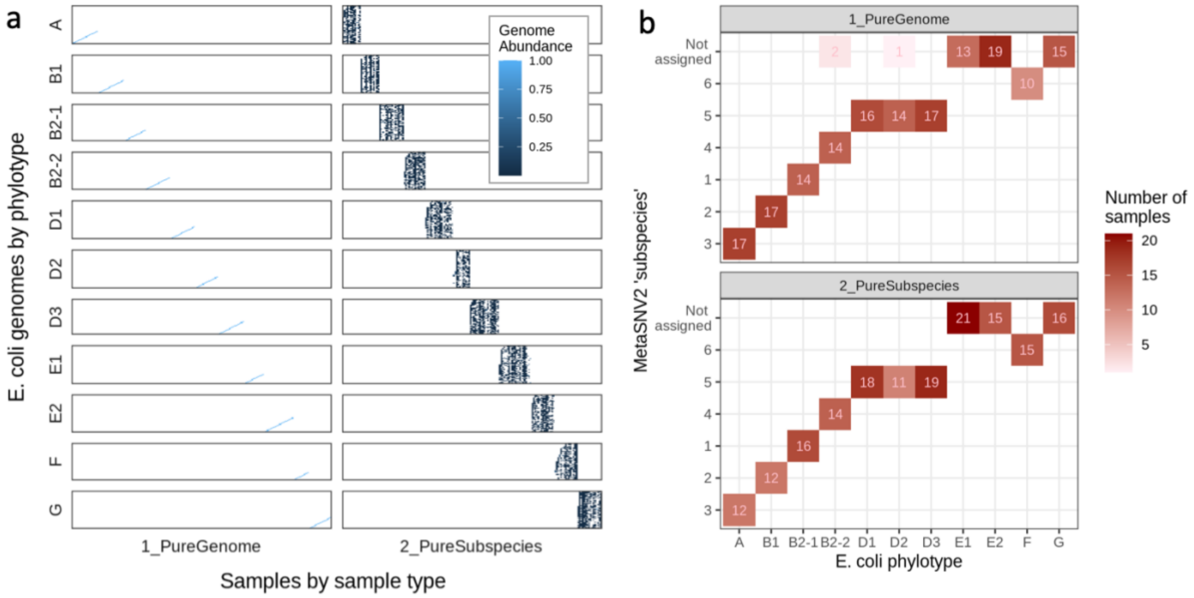

Figure 6. metaSNV v2 assignment of Escherichia coli genomes to known phylotypes. Samples generated *in silico* from single or mixed *E. coli* genomes were classified to subspecies that were identified in this study. (a) Samples (columns) in this test dataset were composed of reads from one *E. coli* genome ("1_PureGenome", N=169) or from multiple genomes belonging to the same phylotype (2_PureSubspecies, N=152). Genomes are rows, grouped by their phylotype. The abundance of each genome in each sample is indicate by the cell colour. (b) Each *E. coli* sample was classified as belonging to a metaSNV v2 subspecies according to the genotyping SNV profile identified in this study (y-axis). This was compared to the previously known phylotype (x-axis). The number of samples with each classification is represented by the colour of each cell and the number written in it. Samples "Not assigned" to a metaSNV v2 subspecies either failed or were not described by the metaSNV v2-generated subspecies scheme due to lack of presence.

The expected phylogroup clades were observed through subspecies calling for 99% of samples and 98% of genomes (318/321 and 166/169, respectively) (**Figure 6b**). Five phylogroups had a one-to-one mapping to the detected subspecies (A, B1, B2-1, B2-2, F), three closely related phylogroups corresponded to one subspecies (D1, D2, D3), and three phylogroups were not reflected in the metaSNV results (E1, E2, G). Phylogroup G is mainly a poultry-associated lineage that can cause extra-intestinal diseases in humans[17], so is not expected to be seen in the human faecal samples studied here. Phylogroup E2 is mainly composed of pathogenic O157:H7 strains[18] and is expected to be present in faecal samples, but only from individuals who are sick, likely with severe, acute diarrhoea and therefore are not likely to be present in this studies' cohort. Phylogroup E2 is most closely related to E1, but a characterisation of typical sources could not be found in literature. Phylogroups that are not prevalent in in this study's cohort cannot contribute to the subspecies model and thus not expected to be reflected in the model. No classification is the correct outcome in these cases.

Validation using B. longum genomes

To assess the accuracy of the identified subspecies, a species with well characterized subspecies was used. Bifidobacterium longum has four subspecies[4] and 12/12 genomes tested were properly categorized based on the genotyping SNVs identified in this study (**Figure 7**). Three *B. longum subsp. infantis* genomes and three *B. longum subsp. longum* genomes were classified to the two subspecies seen in this dataset and the six genomes from the two other subspecies were not classified due to the subspecies not being present in the study dataset.

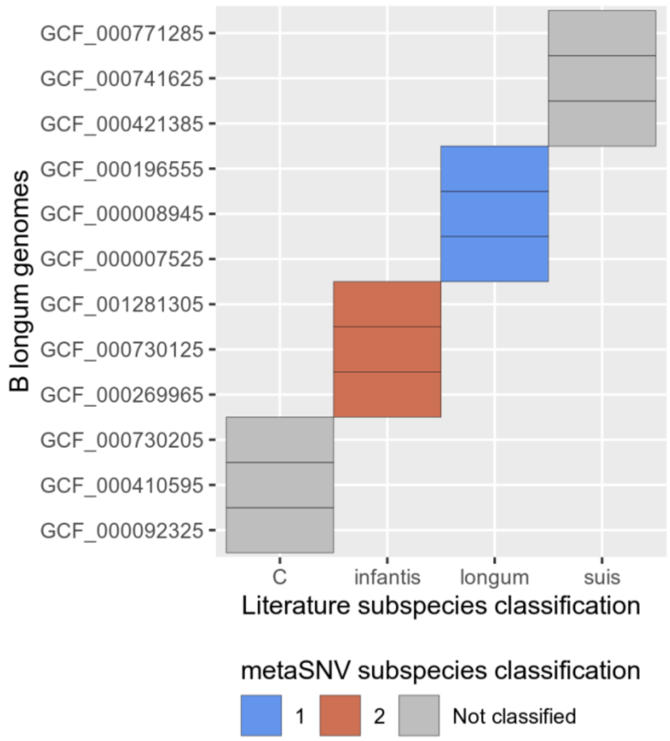

Figure 7. metaSNV v2 assignment of Bifidobacterium longum genomes to established subspecies. Genomes from B longum which are known to belong to different subspecies were classified using the metaSNV v2 subspecies genotyping SNV results. Genomes that were not classified come from subspecies that were not detected in the study dataset.

Subspecies enrichment across age and geography

Many species with subspecies showed an enrichment in at least one country (29/42), and some showed an enrichment with at least one age category (12/42) (**Figure 8**). Of these statistically significant enrichments, only two subspecies had high confidence in the number of clusters and large effect sizes in infants: Bifidobacterium longum and Streptococcus thermophilus (**Figure 9**). Westernised countries tended to have more subspecies in common, and many associations were seen with the Chinese subjects.

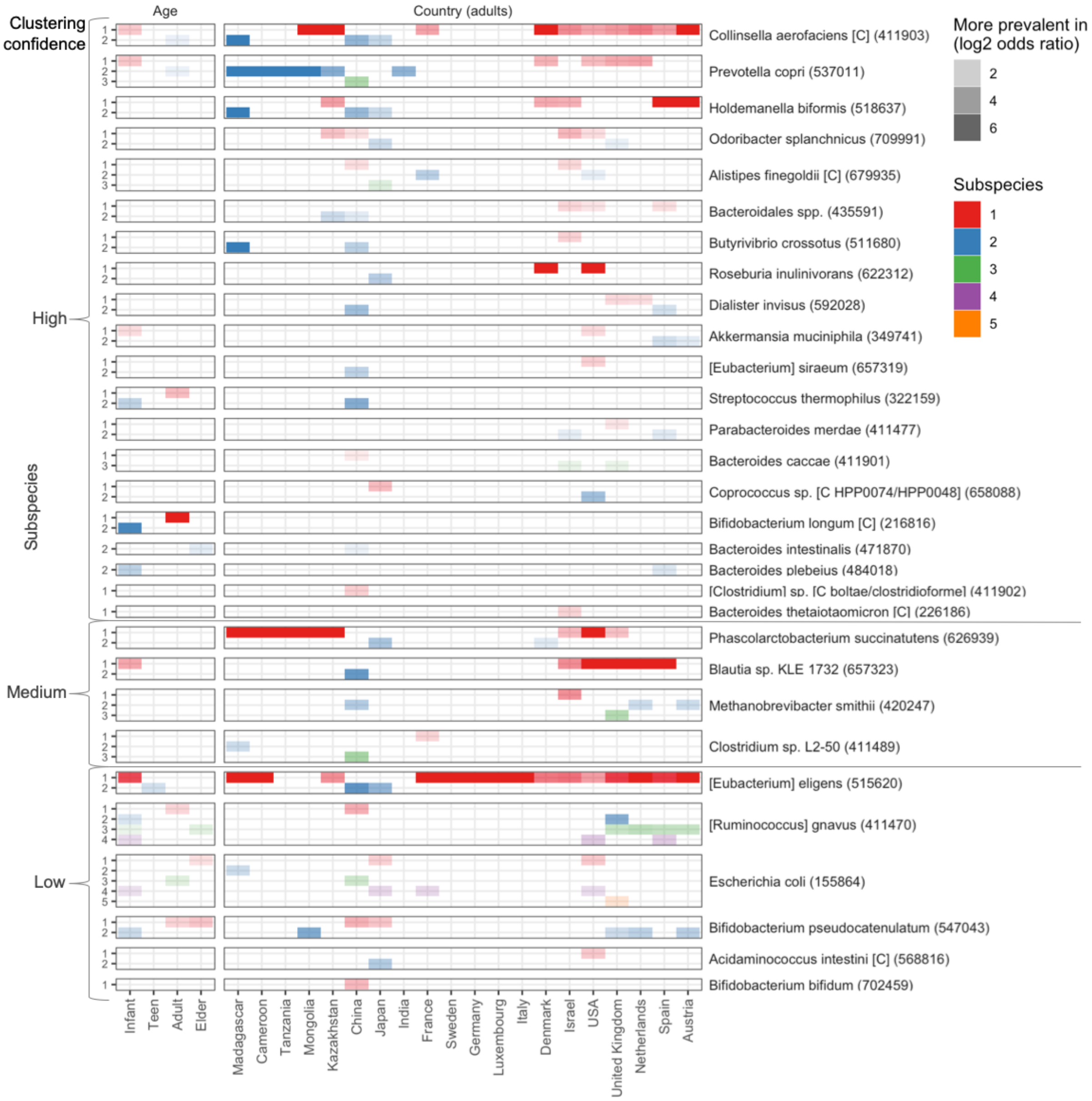

Figure 8. Enrichment of subspecies with host age and location. Many species had enrichment with countries. Only subspecies with at least one statistically significant enrichment are shown. Medium and low confidence subspecies overlaid with grey and demarcated with horizontal lines. Species are arranged by confidence level and then by the number of host associations detected. Countries are arranged by their enrichment similarity (complete clustering on Euclidean distances).

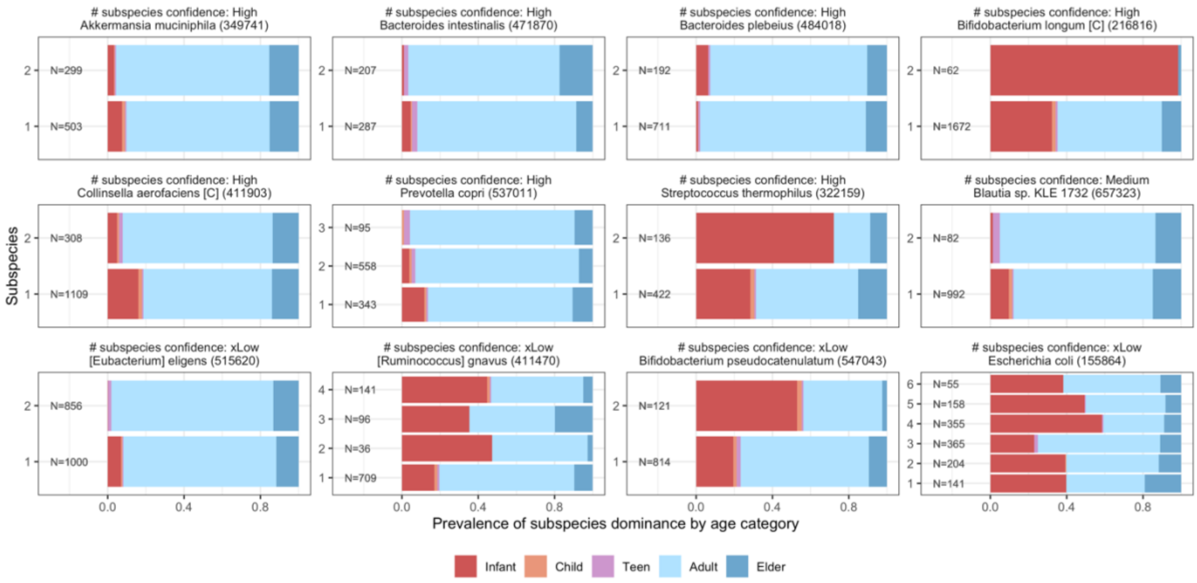

Figure 9. Enrichment of subspecies with age groups. Illustration of the statistically significant age group enrichments shown in Figure 8. Only subjects where one subspecies was dominant (>90%) are shown. The number of samples for each subspecies is indicated as N. The proportion of samples within each subspecies (x axis) that come from each age category are coloured accordingly. Subjects from countries that are only represented by one age group are excluded.

Subspecies gene content

Genes from all polytypic species' pangenomes[13] were tested for correlation with species and subspecies abundances. The genes correlated with species or with subspecies were almost all from the corresponding species' pangenomes (respectively, only 4 out of 30403 and 3 out of 1777 correlated genes were from a different species with a different NCBI species name). Subspecies-specific genes were detected in 24 species and species-specific genes were detected in 30 species out of 30 species tested (**Suppl. File S5**).

When comparing genes from species that had at least 10 species-specific or subspecies-species genes, subspecies-specific genes were enriched for genes with unknown functions, which are more likely to have specific functions rather than broad "housekeeping" functions (**Figure 10**).

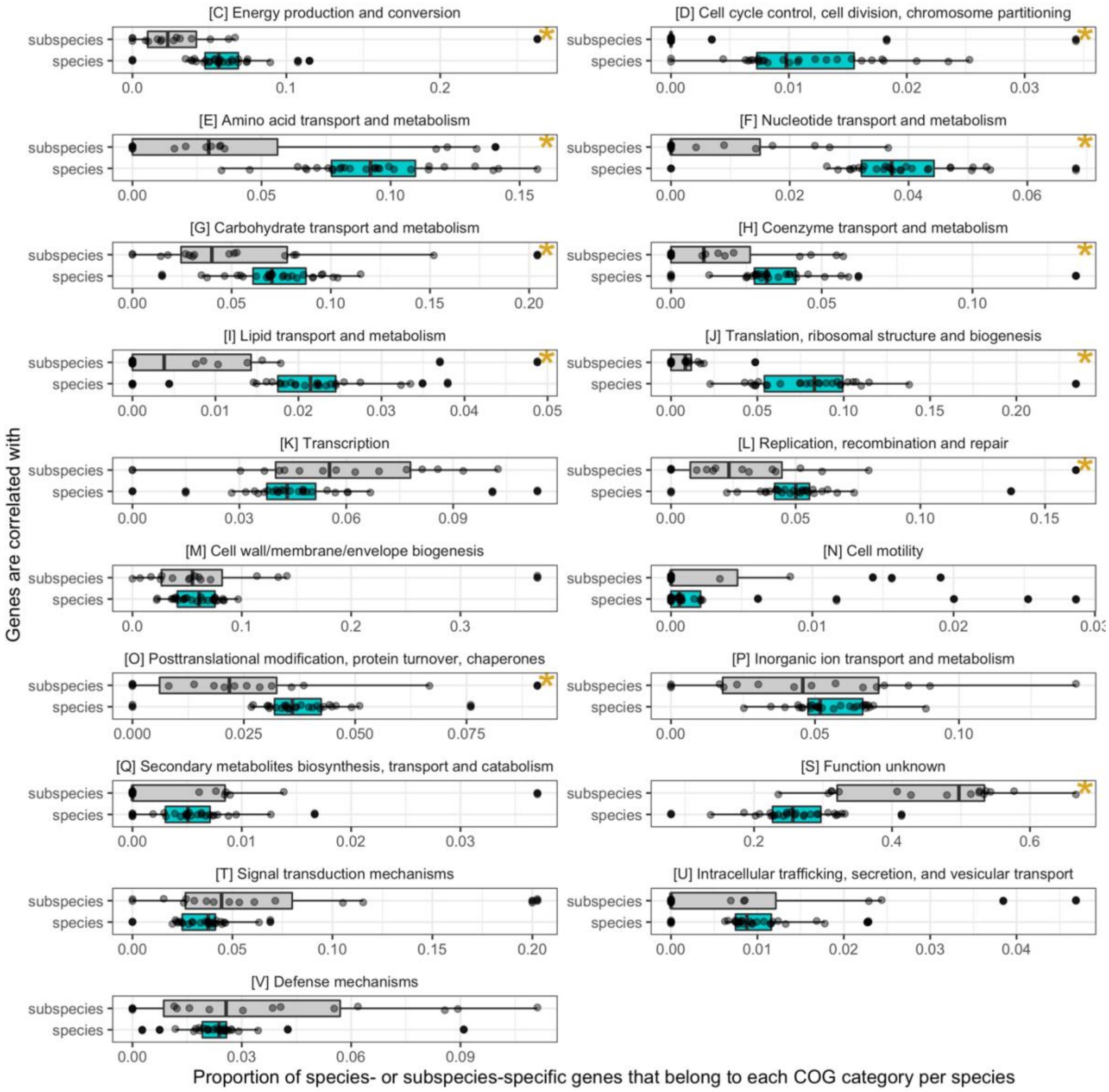

Figure 9. Functional enrichment of subspecies- versus species-specific genes. Values on the x axis are the proportion of species- or subspecies-specific genes that are in each COG category, per species. Stars indicate adjusted p value < 0.05. Species are only used in this analysis if they had at least 10 genes correlated with the species or 10 genes correlated with their subspecies (N=28 species with species-specific genes; N=16 species with subspecies-specific genes).

Impact of population sample on subspecies detection

To illustrate the impact of age and geographic diversity within the sample population on the detection of subspecies and to compare our results with a previous study[7] that took a similar approach, we performed the same analyses described above with a “reduced” dataset. In this “reduced” dataset, we used only adult samples and only samples from 9 countries (Austria, China, Denmark, France, Germany, Kazakhstan, Spain, Sweden, USA). When comparing results between metaSNV v2 analyses on different datasets, a flex in clustering threshold was allowed to avoid noise due to cut-offs (see Methods).

Compared with the full dataset, 85% of species (48/53) had the same monotypic/polytypic classification and 81% (43/53) of species had the same number of subspecies (**Suppl. File S6**). Eight

species had fewer clusters in the reduced dataset and one species had more clusters (**Suppl. File S7** for full results from reduced dataset). For example, *Bifidobacterium longum* and *Streptococcus thermophilus* did not have any clusters in the “reduced” adult-only dataset nor in Costea et al.’s results but had two clusters in the full dataset. This is due to the addition of an infant-enriched cluster for each species. Similarly, *Collinsella aerofaciens* and *Alistipes putredinis* had more clusters in the larger dataset and had diverse geographic enrichment. The one species that had fewer clusters in the larger dataset (*Eubacterium ventriosum*) appears to be due to additional samples bridging the space between the former two clusters, which were also seen in Costea et al.

Overall, in this study’s reduced dataset compared to Costea et al., 87% of species (47/54) had the same monotypic/polytypic classification and 81% (44/54) of species had the same number of subspecies (**Suppl. File S6**), with the mean difference in the number of clusters being -1.3 (maximum -3).

Discussion

Comparison to subspecies in literature

Due to their utility as a level of taxonomic resolution, subspecies have been characterized in some well-studied species. The subspecies and genotyping SNVs identified in this study recapitulated the known subspecies of *Escherichia coli* and *Bifidobacterium longum* and correctly classified isolate genomes that have previously established subspecies classifications. Additionally, a sialidase gene, which is used as a qPCR marker for the *B. longum subsp. infantis* subspecies[21], was observed as subspecies-specific gene, as expected.

Prevotella copri has recently been described to have infraspecific clades, however, the inter-clade genomic dissimilarity is so high (13.0% to 21.4% average nucleotide identity) that these could be considered separate species[22]. Due to the low similarity between these clades and the, generally, species-appropriate mapping cutoff used in this study (97% percent identity), the only clade which was profiled in this study was “Clade A”[22], to which the ProGenomes2 *P. copri* reference genome belongs. Within this clade, we observed a subspecies that is enriched in Chinese subjects, which agrees with the observation of Chinese-associated clades in the phylogenetic tree proposed for *P. copri*’s “Clade A”[22]. *Eubacterium rectale* also has recently described subspecies[23] and was also found to have subspecies in this study, but genotyping SNVs could not be identified, which precluded exact comparisons. The number of subspecies identified for *E. rectale* (four) matches previous reports[23].

The overall finding that roughly half of the tested subspecies in the human gut microbiome have subspecies holds from previous work[7]. When using the full dataset and the “reduced” dataset, the majority of results from this study agree with the results from Costea et al. [7], however some did not. This could be due to the differences in genomic reference sequences used (ProGenomes[24] versus ProGenomes2[9]), the differences in samples used, and small differences in the subspecies detection methodology. The subspecies-specific gene category enriched in this study (“(S) Function unknown”) was also reported by Costea et al[7].

Subspecies in Streptococcus thermophilus

The results presented in this study suggest that *Streptococcus thermophilus* has one subspecies that is found in adults and infants and one that is mostly found in infants. No subspecies of this species have been described in literature, possibly due to lack of study of infant populations. The vast majority (if not all) isolated genomes for this species come from food, mostly fermented milk products, or adult faecal samples (PATRIC DB). These food-derived strains are also seen in human faecal samples and transmission from food to adult gut has been supported by recent large-scale analyses[25]. It is not known from where this species originates in infants. It has not been shown whether maternal food-

derived gut strains are vertically passed to infants (e.g. from faeces, (areolar) skin, breastmilk, and/or gut (e.g. hypothesized "enteromammary" transfer)) or if the populations in infants have a distinct source, such as from the environment. It is not clear from previous work whether mothers harbour different subpopulations of *S thermophilus* in their gut versus in breastmilk. Subspecies specific genes were detected in this study only for the adult enriched subspecies of *S thermophilus*. This may be due to the composition of the pangenome data for *S thermophilus*, from which gene profiles were compiled, because it was only constructed from genomes available, which were isolated from food (mostly fermented milk products).

Effect of sample population

The comparison between the full and the reduced datasets, with narrower geography and age ranges, demonstrates how the results of subspecies analysis depend on the input population. Some subspecies were added in the full dataset, either due to addition of separate subspecies or due to additional samples reinforcing existing clustering. For example, *Bifidobacterium longum* was not detected to have subspecies in the reduced dataset or in a previous adults-only analysis[7], but its subspecies known to be associated with infants (*ssp. infantis*) was detected in the dataset which included infants. With a larger dataset, addition of clusters was more common than merging of clusters, but both cases were seen.

Limitations

As with any study, a major limitation here can be due to the composition of the input data. We cannot see what we do not sample. Subspecies can only be detected if they are in the subpopulations sampled, and therefore subspecies may or may not be detected given the sample population make up, such as country or age. This was demonstrated by the difference in results between the full and the "reduced" datasets. The presence of more diverse samples can increase the cluster number of some species by adding new subspecies, e.g. from infants or from additional countries, but can also decrease the number of clusters by bridging two clusters. In this study, the former was more common. Even the presence of additional samples from the same subspecies can increase the number of clusters by reinforcing existing clusters that previously had too few samples to be detected robustly. Whether enough samples were input for metaSNV v2 to make a robust claim on the number of subspecies is estimated from rarefaction and output as the "cluster number confidence" classifications of high, medium, or low. The majority of subspecies number classifications in this study were "high". There is always the possibility that more subspecies exist than detected from metaSNV v2 results, as sampling of a global population is very rarely, if ever, complete.

Two main limitations are also intrinsic to the methodological approach of metaSNV v2. First, the subpopr module relies of the existence of many metagenomic samples where one subspecies is very dominant (e.g. more than 50 samples where a subspecies is >90% abundant, by default). Second, the SNV calls are based on reference genomes. If the default suggested mapping parameters are used (minimum match of 97% identity), then metaSNV can only detect SNVs in sections of the genome that have a typical within-species similarity. This means that for unusually divergent species complexes, such as Prevotella copri[22], the full range of the sample population cannot be covered.

This analysis could be extended in a few ways. The number of species studied could be increased. If the species studied were expanded to the 160 species that had an abundance greater than 0.1% in at least 200 samples, then the median total relative community abundance represented would be 69%, which is an increase of 18 percentage points over the current set of 71 species. The gene content analysis was based on gene profiles from pangenomes, which were built based on public reference genomes. This means that only genes that come from an isolate sequence can be detected and novel associations are less likely to be detected. Gene profiles compiled from a gene catalogue, such as the IGC or the GMGC would allow a broader perspective of subspecies gene content, but requires substantially more computational time due to the large sizes of the databases.

Conclusions

With a more diverse and fourfold larger dataset, we present more evidence to support the previous estimate that overall roughly half of the tested subspecies in the human gut microbiome have subspecies. Detection of subspecies is sensitive to population samples, and it is likely that with more sampling, especially of traditionally understudied populations, more subspecies will be discovered. While many species are found in both infant and adult faecal microbiomes, subspecies being distinctly associated with either age group is uncommon. Two instances were observed in this study, one of which is well known, *B longum*, and one of which provides an avenue for future study, *S thermophilus*. The strategy and tool presented above can be used to study subspecies in other microbiome populations.

References

1. Monroe B. A Modern Concept of the Subspecies. AOS, editor. Auk. 1982;99:608–9.

2. Patten MA. Subspecies and the philosophy of science. Auk [Internet]. Narnia; 2015 [cited 2019 Jul 25];132:481–5. Available from: https://academic.oup.com/auk/article/132/2/481-485/5149032

3. Van Rossum T, Ferretti P, Maistrenko OM, Bork P. Diversity within species: interpreting strains in microbiomes. Nat. Rev. Microbiol. 2020.

4. O'Callaghan A, Bottacini F, O'Connell Motherway M, van Sinderen D. Pangenome analysis of Bifidobacterium longum and site-directed mutagenesis through by-pass of restriction-modification systems. BMC Genomics. 2015;

5. Bailey JK, Pinyon JL, Anantham S, Hall RM. Distribution of human commensal Escherichia coli phylogenetic groups. J Clin Microbiol [Internet]. American Society for Microbiology Journals; 2010 [cited 2019 May 3];48:3455–6. Available from: http://www.ncbi.nlm.nih.gov/pubmed/20610687

6. Mayr E. Populations, Species, and Evolution. An Abridgment of Animal Species and Evolution [Internet]. Harvard University Press; 1970. Available from: https://www.jstor.org/stable/3241850?origin=crossref

7. Costea P, Coelho LP, Sunagawa S, Munch R, Huerta-Cepas J, Forslund K, et al. Subspecies in the global human gut microbiome. Mol Syst Biol [Internet]. EMBO Press; 2017 [cited 2018 Jan 4];13:960–960. Available from: http://www.ncbi.nlm.nih.gov/pubmed/29242367

8. Milanese A, Mende DR, Paoli L, Salazar G, Ruscheweyh H-J, Cuenca M, et al. Microbial abundance, activity and population genomic profiling with mOTUs2. Nat Commun [Internet]. Nature Publishing Group; 2019 [cited 2019 May 3];10:1014. Available from: http://www.nature.com/articles/s41467-019-08844-4

9. Mende DR, Letunic I, Maistrenko OM, Schmidt TSB, Milanese A, Paoli L, et al. ProGenomes2: An improved database for accurate and consistent habitat, taxonomic and functional annotations of prokaryotic genomes. Nucleic Acids Res. 2020;

10. Li H, Durbin R. Fast and accurate short read alignment with Burrows-Wheeler transform. Bioinformatics. 2009;

11. Coelho LP, Alves R, Monteiro P, Huerta-Cepas J, Freitas AT, Bork P. NG-meta-profiler: Fast processing of metagenomes using NGLess, a domain-specific language. Microbiome. 2019;

12. Li H, Handsaker B, Wysoker A, Fennell T, Ruan J, Homer N, et al. The Sequence Alignment/Map format and SAMtools. Bioinformatics. 2009;

13. Maistrenko OM, Mende DR, Luetge M, Hildebrand F, Schmidt TSB, Li SS, et al. Disentangling the impact of environmental and phylogenetic constraints on prokaryotic within-species diversity. ISME J [Internet]. Cold Spring Harbor Laboratory; 2020 [cited 2019 Oct 23];1:735696. Available from: https://www.biorxiv.org/content/10.1101/735696v1

14. Huerta-Cepas J, Szklarczyk D, Heller D, Hernández-Plaza A, Forslund SK, Cook H, et al. EggNOG 5.0: A hierarchical, functionally and phylogenetically annotated orthology resource based on 5090 organisms and 2502 viruses. Nucleic Acids Res. 2019;

15. R Core Team. R: A language and environment for statistical computing [Internet]. Vienna, Austria;

2013. Available from: http://www.r-project.org/

16. Benjamini Y, Hochberg Y. Controlling the false discovery rate: a practical and powerful approach to multiple testing. J R Stat Soc Ser B [Internet]. 1995 [cited 2013 Sep 25];57:289–300. Available from: http://www.jstor.org/stable/10.2307/2346101

17. Clermont O, Dixit OVA, Vangchhia B, Condamine B, Dion S, Bridier-Nahmias A, et al. Characterization and rapid identification of phylogroup G in *Escherichia coli* , a lineage with high virulence and antibiotic resistance potential. Environ Microbiol [Internet]. Blackwell Publishing Ltd; 2019 [cited 2021 Apr 9];21:3107–17. Available from: https://onlinelibrary.wiley.com/doi/abs/10.1111/1462-2920.14713

18. Abram K, Udaondo Z, Bleker C, Wanchai V, Wassenaar TM, Robeson MS, et al. Mash-based analyses of Escherichia coli genomes reveal 14 distinct phylogroups. Commun Biol [Internet]. 2021;4:117. Available from: http://www.nature.com/articles/s42003-020-01626-5

19. Waters NR, Abram F, Brennan F, Holmes A, Pritchard L. Easy phylotyping of Escherichia coli via the EzClermont web app and command-line tool. Access Microbiol [Internet]. Microbiology Society; 2020 [cited 2021 Apr 9];2:e000143. Available from: https://www.microbiologyresearch.org/content/journal/acmi/10.1099/acmi.0.000143

20. Ondov BD, Treangen TJ, Melsted P, Mallonee AB, Bergman NH, Koren S, et al. Mash: Fast genome and metagenome distance estimation using MinHash. Genome Biol [Internet]. Genome Biology; 2016;17:029827. Available from: http://biorxiv.org/content/early/2015/10/26/029827.abstract

21. Lawley B, Munro K, Hughes A, Hodgkinson AJ, Prosser CG, Lowry D, et al. Differentiation of *Bifidobacterium longum* subspecies *longum* and *infantis* by quantitative PCR using functional gene targets. PeerJ [Internet]. PeerJ Inc.; 2017 [cited 2018 Jul 3];5:e3375. Available from: https://peerj.com/articles/3375

22. Tett A, Huang KD, Asnicar F, Fehlner-Peach H, Pasolli E, Karcher N, et al. The Prevotella copri Complex Comprises Four Distinct Clades Underrepresented in Westernized Populations. Cell Host Microbe. 2019;

23. Karcher N, Pasolli E, Asnicar F, Huang KD, Tett A, Manara S, et al. Analysis of 1321 Eubacterium rectale genomes from metagenomes uncovers complex phylogeographic population structure and subspecies functional adaptations. Genome Biol [Internet]. BioMed Central; 2020 [cited 2020 Jul 23];21:138. Available from: https://genomebiology.biomedcentral.com/articles/10.1186/s13059-020-02042-y

24. Mende DR, Letunic I, Huerta-Cepas J, Li SS, Forslund K, Sunagawa S, et al. proGenomes: a resource for consistent functional and taxonomic annotations of prokaryotic genomes. Nucleic Acids Res [Internet]. Oxford University Press; 2017 [cited 2019 May 3];45:D529–34. Available from: http://www.ncbi.nlm.nih.gov/pubmed/28053165

25. Pasolli E, De Filippis F, Mauriello IE, Cumbo F, Walsh AM, Leech J, et al. Large-scale genome-wide analysis links lactic acid bacteria from food with the gut microbiome. Nat Commun. 2020;

26. Kim HB, Kim E, Yang SM, Lee S, Kim MJ, Kim HY. Development of Real-Time PCR Assay to Specifically Detect 22 Bifidobacterium Species and Subspecies Using Comparative Genomics. Front Microbiol. 2020;
