# Supplementary material for: metaSNV v2: detection of SNVs and subspecies in prokaryotic metagenomes
Source: Bioinformatics. 2021 Nov 17;38(4):1162–4. doi: 10.1093/bioinformatics/btab789 (PMC8796361; doi:10.1093/bioinformatics/btab789)
Supplement: btab789_supplementary_data [file btab789_supplementary_data.zip › S2_Ocean.pdf]

This document describes the results of an analysis run with metaSNV v2.

Subspecies detection in oceanic samples

Ocean metagenomes from the TARA Oceans sampling expedition[1] (N=288) were tested for the presence of subspecies. Metagenomes from the deep chlorophyll maximum (DCM, N=98), the mesopelagic zone (MES, N=49), and surface water (SUR, N=132) were used. These samples were collected globally from 2009/09/15 to 2012/03/19 (Figure 1).

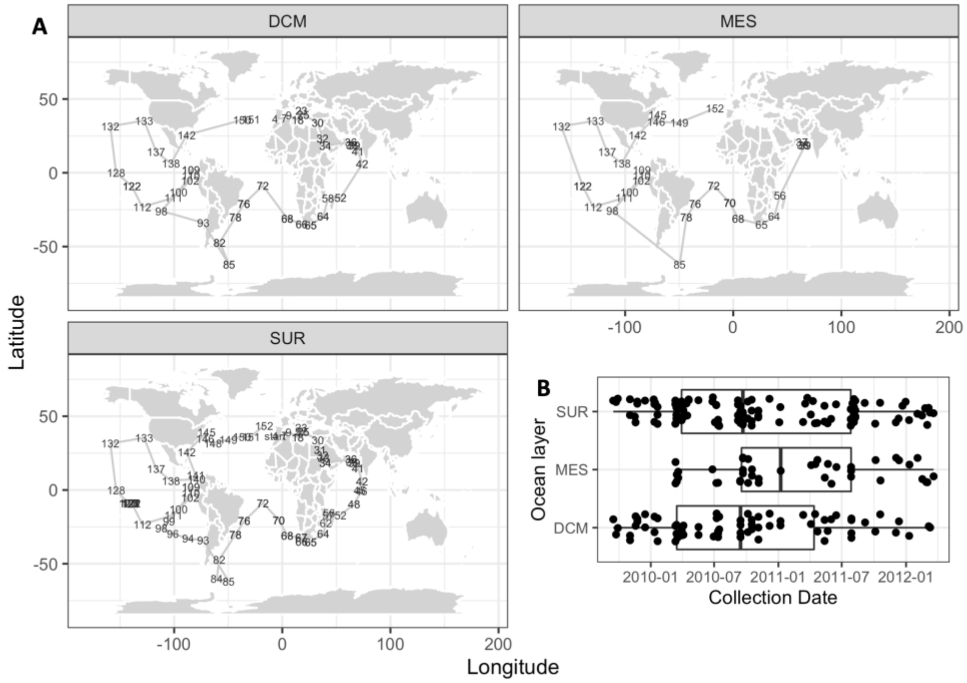

Figure 1. Ocean sampling sites. Sampling sites across space (A) and time (B). Numbers on maps are sampling site IDs (N=288). Lines connect the shortest distance between subsequent sampling points and do not depict the route travelled. The starting point was in the Mediterranean Sea. Subplots show samples collected at different depths: the deep chlorophyll maximum (DCM, N=98), the mesopelagic zone (MES, N=49), and surface water (SUR, N=132). Points in the box plot are randomly spread on the y axis for visibility.

Subspecies were profiled by mapping metagenomic short reads against the ProGenomes2 database of species representative bacterial genomes[2]. Mappings were performed using bwa[3] and ngless[4]. Only reads that mapped uniquely with at least 97% identity to the species reference were kept. Mappings were analyzed using metaSNV v2 to identify SNVs and subspecies. Only metagenomes with at least

a depth of coverage of 5 bp across 40% of the reference genome were used. Only SNV positions that had at a minimum vertical coverage of 5 per sample in at least 50% of samples were kept. Species were only analyzed if they had data from at least 50 metagenomes. This resulted in 1,901,938 SNVs (51,422 to 273,454 per species) identified across 184 samples and 10 species. Defaults for all parameters were used, except that the minimum number of metagenomes per species was lowered from 100 to 50 to due to the smaller total sample size.

Two species showed evidence of having subspecies: SAR86 cluster bacterium SAR86A (NCBI taxon ID 1123866) (Figure 2) and candidatus *Pelagibacter ubique* (NCBI taxon ID 1096769) (Figure 3). Both subspecies showed geographic enrichment in the South Pacific Ocean, which was sampled from March to September 2011, and were observed mainly in the DCM and SUR ocean layers.

SAR86 is a ubiquitous order of marine bacteria that is known to have spatiotemporal ecotypes[5]. According to the GTDB SAR86A is a genus level clade within this order containing multiple species. Despite this genus level classification, these subpopulations likely still represent subspecies because only reads that mapped uniquely to the reference genome with at least 97% identity were used in this analysis. This threshold is generally recognised as a species boundary[6].

Pelagibacter ubique is a species according to the GTDB, but other species exist that are named similarly, e.g. *Pelagibacter ubique* A, *Pelagibacter ubique* B etc. ProGenomes2 has seven species called "*Pelagibacter ubique*". Despite this naming system, the subpopulations identified here likely still represent subspecies, following the same reasoning as with SAR86A described above. A species of *Pelagibacter ubique,* which is not observed in the South Pacific, has been found to have gene content clusters that are geographically enriched[7].

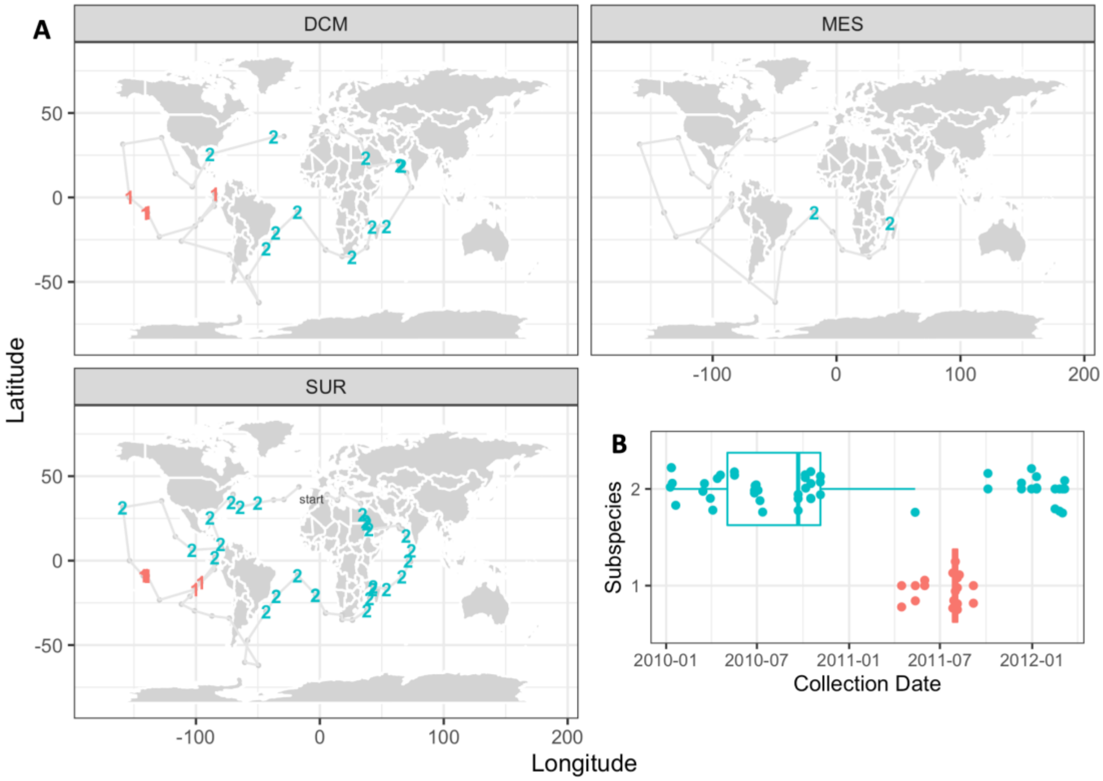

Figure 2. Subspecies in SAR86 sp. Subspecies were observed in distinct (A) geographic and/or (B) temporal niches. Red "1"s are samples where subspecies 1 dominated, blue "2"s are samples where subspecies 2 dominated. Lines connect the shortest distance between subsequent sampling points and do not depict the route travelled. Points in the box plot are randomly spread on the y axis for visibility.

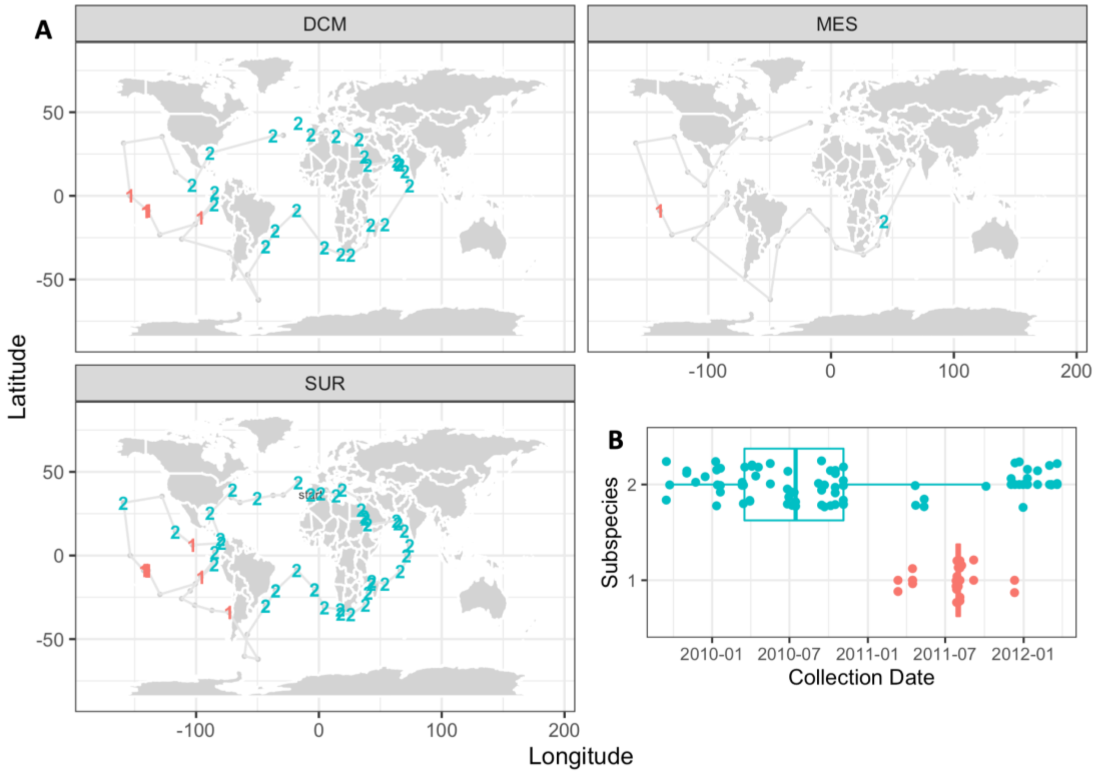

Figure 3. Subspecies in candidatus *Pelagibacter ubique* species. Subspecies were observed in distinct (A) geographic and/or (B) temporal niches. Red "1"s are samples where subspecies 1 dominated, blue "2"s are samples where subspecies 2 dominated. Lines connect the shortest distance between subsequent sampling points and do not depict the route travelled. Points in the box plot are randomly spread on the y axis for visibility.

1. Sunagawa S, Coelho LP, Chaffron S, Kultima JR, Labadie K, Salazar G, et al. Structure and function of the global ocean microbiome. Science (80-). 2015;348:1261359–1261359. doi:10.1126/science.1261359.
2. Mende DR, Letunic I, Maistrenko OM, Schmidt TSB, Milanese A, Paoli L, et al. ProGenomes2: An improved database for accurate and consistent habitat, taxonomic and functional annotations of prokaryotic genomes. Nucleic Acids Res. 2020.
3. Li H, Durbin R. Fast and accurate short read alignment with Burrows-Wheeler transform. Bioinformatics. 2009.
4. Coelho LP, Alves R, Monteiro P, Huerta-Cepas J, Freitas AT, Bork P. NG-meta-profiler: Fast processing of metagenomes using NGLess, a domain-specific language. Microbiome. 2019.
5. Hoarfrost A, Nayfach S, Ladau J, Yooseph S, Arnosti C, Dupont CL, et al. Global ecotypes in the ubiquitous marine clade SAR86. ISME J. 2020;14:178–88. doi:10.1038/s41396-019-0516-7.
6. Jain C, Rodriguez-R LM, Phillippy AM, Konstantinidis KT, Aluru S. High throughput

ANI analysis of 90K prokaryotic genomes reveals clear species boundaries. Nat Commun. 2018;9:5114.
7. Scholz M, Ward D V., Pasolli E, Tolio T, Zolfo M, Asnicar F, et al. Strain-level microbial epidemiology and population genomics from shotgun metagenomics. Nat Methods. 2016;13:435–8.
