# Supplementary material for: metaSNV v2: detection of SNVs and subspecies in prokaryotic metagenomes
Source: Bioinformatics. 2021 Nov 17;38(4):1162–4. doi: 10.1093/bioinformatics/btab789 (PMC8796361; doi:10.1093/bioinformatics/btab789)
Supplement: btab789_supplementary_data [file btab789_supplementary_data.zip › S3_Ecoli.pdf]

Validation of metaSNV v2 results using *in silico* samples made from *E. coli* genomes belonging to known subspecies and comparison with StrainPhlAn

Design of in silico mock communities

E coli genomes can be classified into to phylogroups[1], which are analogous to subspecies. Here, we used this known structure to demonstrate how the metaSNV v2 subspecies module ("subpopr") can recover underlying population structure from metagenomic data.

There are two main approaches to classify E coli genomes into phylogroups: Clermont typing based on marker gene presence, which generally recognises 7 phylogroups, and whole genome based[2], which recognises 17 phylogroups. This difference is both from subdivision and addition. For example, the genome method breaks up type "B2" into "B2-1" and "B2-2", and type "D" into "D1", "D2", and "D3", and it also has extra groups, such as "Shigella1" and "Shigella2". For our analysis, we used genomes where both methods agreed on the phylogroup classification. Classifications were used from published data[2], where they were made using EzClermont[3] and Mash[4].

For each sample, *E coli* genome abundances were designed to reflect one community type:

1. "1_PureGenome": Reads from 1 genome
2. "2_PureSubspecies": Reads from multiple genomes from the same phylogroup
3. "3_ImpureSubspecies": Reads from multiple genomes from multiple phylogroups with one phylogroup being dominant
4. "4_MixSubpecies": Reads from multiple genomes from multiple phylogroups evenly abundant

To create these samples, we simulated reads with 20x depth of coverage from 540 *E coli* genomes (46 to 50 per phylogroup, based on availability), then sampled a defined number of reads from each genome's read set according to the genome's abundance in the simulated community (Figure 1a). This created 543 simulated metagenomic samples (columns in Figure 1a).

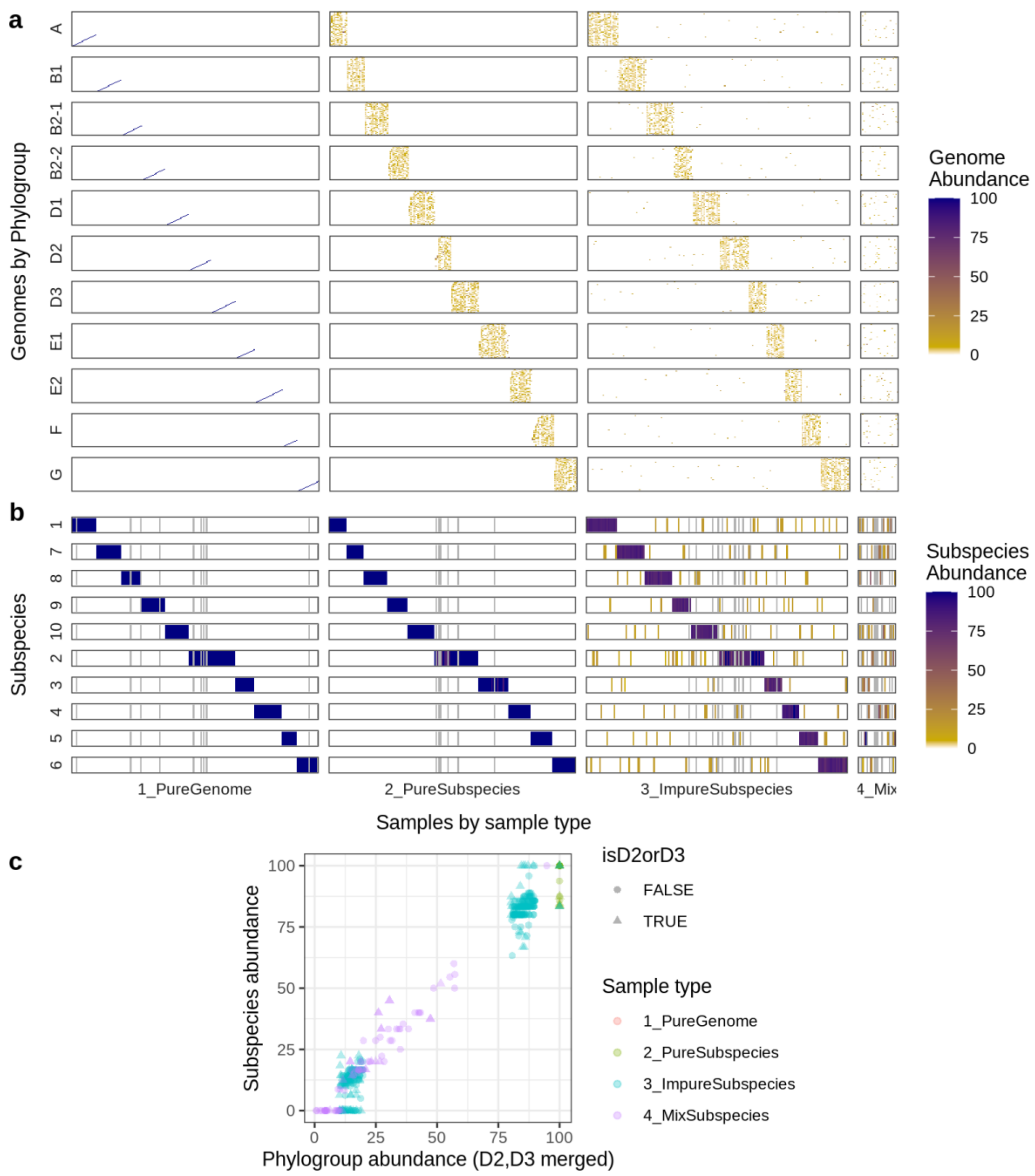

Figure 1. Abundance of phylogroups and subspecies in mock community *E coli* samples. The genome composition of each sample is depicted in (a), where each row is a genome that belongs to a phylogroup (N=540). Every column is a sample (N=543) made up of reads simulated from one or more *E coli* genomes. The columns in (a) correspond to the columns in (b) (are the same samples). The rows in (b) are the subspecies called by metaSNV v2 (1 to 10) and the colours reflect the abundance of each subspecies in each sample. Subspecies 1 corresponds to Phylogroup A (and 7 to B1, 8 to B2-1, etc.). The grey colour indicates a sample did not the meet criteria for inclusion in the subspecies profiling due to low coverage or to lack of genotyping SNVs (D2). The phylogroup and subspecies abundances per sample are plotted against each other in (c), where every point is a sample-subspecies/phylogroup pair. The data points from “1_PureGenome” and “2_PureSubspecies” are over-plotted, with most values at 1 for both phylogroup and subspecies abundances. Since most samples from

D2 and D3 were combined into one output subspecies, the abundances of these subspecies and phylogroups were combined in this analysis.

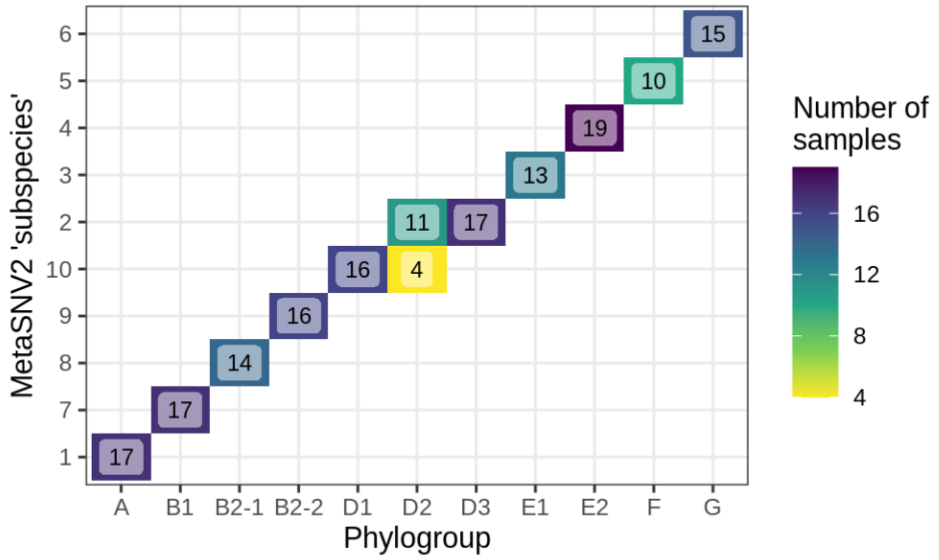

Figure 2. Correspondence of phylogroups and subspecies of genomes in mock community *E coli* samples. Samples shown here are a subset of the total set (N=169). These samples were composed of reads generated in silico from one E coli genome each (type “1_PureGenome”). These genomes have previously characterised phylotypes. Each cell represents the number of samples that belong to each phylotype (x axis) and that were assigned to each metaSNV v2 subspecies (y axis). For example, all 17 samples composed of reads from genomes that belong to phylotype A were assigned to subspecies 1.

Detection of SNVs and subspecies

The method to detect SNVs and subspecies used here is the same as used in the human study described in Suppl. Mat. 2.

We mapped the reads from the *in silico* generated samples against the default metaSNV reference *E coli* genome as well as 69 other species’ genomes using BWA and ngless with a minimum alignment length of 45bp and a minimum identity of 97%. Only uniquely mapped reads were kept.

SNVs were called from these mappings using metaSNV v2 (metaSNV.py). Resultant SNVs were filtered with default parameters (metaSNV_Filtering.py -b 40 -d 5 -m 2 -c 5 -p 0.5) to remove low prevalence and low abundance SNVs. Distances between samples were calculated using Manhattan distance based on the SNV profiles (metaSNV_DistDiv.py). Subspecies were called using default parameters (metaSNV_subpopr.R -x 0.1 -y 0.8 -z 0.8).

Sample classifications were obtained from the original clustering/subspecies results and subspecies abundances were calculated based on the abundance of genotyping SNVs (profileSamplesUsingGenotypes.R).

Comparison of detected subspecies to original phylogroups

The underlying population structure constructed in the mock community dataset (Figure 1a) was recovered through subspecies calling (Figure 1b, Figure 2). All phylogroups had a one-to-one mapping to the detected subspecies except the D phylogroups. Some genomes from the D2 phylogroup could not be distinguished with this approach from the closely related groups D1 and D3 (Figure 2, Figure 1b). If the D2 and D3 phylogroups are considered as one, then genomes were accurately classified to their phylogroups in 98% of cases (165/169, Figure 2). The correlation was high between the phylogroup abundances, which were set when making the *in silico* samples, and the corresponding subspecies abundances, which were based on the abundances of genotyping SNVs identified and profiled by metaSNV v2 (Pearson R 0.99, $p < 2.2e-16$; Spearman R 0.94 $p < 2.2e-16$) (Figure 1c).
Missing subspecies abundance data (Figure 2b) is due to low coverage in the *in silico* data or unusually dissimilar genomes.

Impact of subspecies detection parameter choice

Varying the sample-selection parameters (hr & hs) in the subspecies calling module can impact the number of subspecies called (Figure 2). Varying the parameters can impact the selection and thus quantity of samples used to discover clustering (Figure 2a), and lower samples numbers resulted in fewer clusters (Figure 2b). As fewer clusters were identified, more closely related phylogroups were clustered together (Table 1).

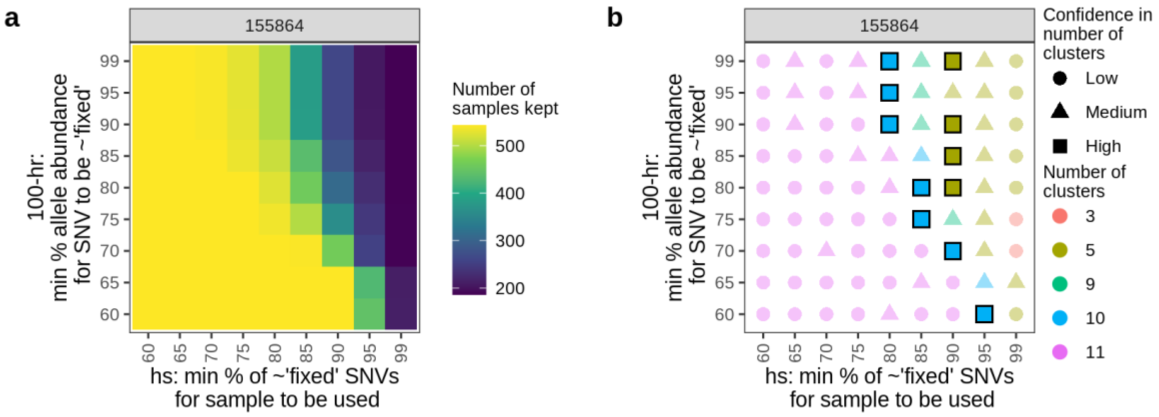

Figure 2. Impact of parameter settings on subspecies module results. The hr and hs parameters control the selection of “discovery” samples used in the subspecies calling process. This selection impacts the number of samples used (a) and the number of subspecies called (b).

<table>
<tr><th>N Clusters</th><th colspan="11">Phylogroup correspondence to subspecies clusters</th></tr>
<tr><td>3</td><td colspan="4">A,B1,E1,E2</td><td colspan="2">B2-1,B2-2</td><td colspan="5">D1,D2,D3,F,G</td></tr>
<tr><td>5</td><td colspan="2">A,B1</td><td colspan="2">E1,E2</td><td colspan="2">B2-1,B2-2</td><td colspan="3">D1,D2,D3</td><td>F</td><td>G</td></tr>
<tr><td>9</td><td>A</td><td>B1</td><td colspan="2">E1,E2</td><td>B2-1</td><td>B2-2</td><td colspan="2">D1,D2</td><td>D2,D3</td><td>F</td><td>G</td></tr>
<tr><td>10</td><td>A</td><td>B1</td><td>E1</td><td>E2</td><td>B2-1</td><td>B2-2</td><td colspan="2">D1,D2</td><td>D2,D3</td><td>F</td><td>G</td></tr>
<tr><td>11</td><td>A</td><td>B1</td><td>E1</td><td>E2</td><td>B2-1</td><td>B2-2</td><td>D1</td><td>D2</td><td>D2,D3</td><td>F</td><td>G</td></tr>
</table>

Table 1. Correspondence between phylotypes and metaSNV v2 subspecies across parameter settings. The same samples were analysed with varying values for parameters "hr" and "hs" (Figure 2), resulting in different numbers of clusters (rows). This table shows how samples from different phylotypes were classified to metaSNV v2 clusters (columns), across clustering results. Each outlined cell reflects one metaSNV v2 cluster. Only samples composed of a single subspecies are used here ("1_PureGenome", "2_PureSubspecies"). This shows that as the cluster number increased, clusters were split consistently into respective phylogroups.

In conclusion, metaSNV v2 was able to accurately recover the expected phylogroups and accurately measure their abundance, except for some genomes from the D2 phylogroup. These genomes did not form their own cluster but instead were grouped with the closely related D1 or D3 phylogroups. When parameter choice decreased the number of subspecies detected, closely related phylogroups were merged. Thus, parameter choice changed the resolution but not the validity of the produced clustering.

Comparison with StrainPhlAn's SNV-based population similarity quantification

Similar to metaSNV, StrainPhlAn[5] calls SNVs in metagenomes per species and provides dissimilarity measures for metagenomic species population pairs based on their SNV profiles. Two metagenomes can thus be compared based on their respective populations of a particular species. StrainPhlAn uses clade-specific marker genes as references for SNV calling, while metaSNV uses whole genomes. StrainPhlAn runs on one species at a time and the resultant sample dissimilarities are not clustered into subspecies. To compare metaSNV 2 and StrainPhlAn, StrainPhlAn was run on the *in silico* generated *E. coli* metagenomes described above and the SNV-profile based metagenome dissimilarities were compared. Subspecies classifications cannot be compared as these are not produced by StrainPhlAn.

StrainPhlAn[6] version 3.0.9 was run with default parameters on the *in silico* generated *E. coli* metagenomes described above. Dissimilarities produced by StrainPhlAn and by metaSNV 2 and were strongly correlated, both when looking at all *in silico* metagenomes (Pearson R=0.75, Spearman R=0.81; N = 537 samples and 143,916 pairwise comparisons; p-value < 2.2e-16 and when looking at those samples composed of only one genome ("1_PureGenome") (Pearson R=0.91, Spearman R=0.85; N =164 samples and 13,366 pairwise comparisons; p-value < 2.2e-16). Differences in dissimilarity values between the two methods are likely due to StrainPhlAn calling SNVs in marker genes and not counting minor alleles when calculating SNV-based dissimilarities, while metaSNV v2 calls SNVs on core genomes and does take minor alleles into account.

1. Clermont, O. *et al.* Characterization and rapid identification of phylogroup G in *Escherichia coli* , a lineage with high virulence and antibiotic resistance potential. *Environ. Microbiol.* **21**, 3107–3117 (2019).
2. Abram, K. *et al.* Mash-based analyses of Escherichia coli genomes reveal 14 distinct phylogroups. *Commun. Biol.* **4**, 117 (2021).
3. Waters, N. R., Abram, F., Brennan, F., Holmes, A. & Pritchard, L. Easy phylotyping of Escherichia coli via the EzClermont web app and command-line tool. *Access*

Microbiol. **2**, e000143 (2020).
4. Ondov, B. D. *et al.* Mash: Fast genome and metagenome distance estimation using MinHash. *Genome Biol.* **17**, 029827 (2016).
5. Truong, D. T., Tett, A., Pasolli, E., Huttenhower, C. & Segata, N. Microbial strain-level population structure and genetic diversity from metagenomes. *Genome Res.* **27**, 626–638 (2017).
6. Beghini, F. *et al.* Integrating taxonomic, functional, and strain-level profiling of diverse microbial communities with biobakery 3. *Elife* **10**, (2021).
